# Novel pennate diatom symbionts support high N_2_ fixation rates

**DOI:** 10.1093/ismeco/ycaf190

**Published:** 2025-10-24

**Authors:** Bhavya S Panthalil, Angela Vogts, Mar Benavides, Matthew J Harke, Christiane Hassenrück, Ajit Subramaniam, Joseph P Montoya, Maren Voss

**Affiliations:** Department of Biological Oceanography, Leibniz Institute for Baltic Sea Research Warnemünde, Rostock 18119, Germany; Department of Biological Oceanography, Leibniz Institute for Baltic Sea Research Warnemünde, Rostock 18119, Germany; National Oceanography Centre, European Way, Southampton SO14 3ZH, United Kingdom; Aix Marseille Univ, Université de Toulon, CNRS, IRD, MIO UM 110, Marseille 13288, France; Turing Centre for Living Systems, Aix-Marseille University, Marseille 13009, France; Fisheries and Ocean Health, Gloucester Marine Genomics Institute, Gloucester, MA 01930, United States; Department of Biological Oceanography, Leibniz Institute for Baltic Sea Research Warnemünde, Rostock 18119, Germany; Lamont-Doherty Earth Observatory at Columbia University, Palisades, NY 10964, United States; School of Biological Sciences, Georgia Institute of Technology, Atlanta, GA 30332, United States; Department of Biological Oceanography, Leibniz Institute for Baltic Sea Research Warnemünde, Rostock 18119, Germany

**Keywords:** nitrogen fixation, symbiosis, diatoms, tropical North Atlantic Ocean, NanoSIMS, cell-specific N_2_ fixation

## Abstract

Diazotrophy is the most important nitrogen source in the oligotrophic surface ocean, but the organisms involved and their contributions are incompletely understood due to limited observations. Only diazotrophic organisms possess the *nifH* gene to reduce dinitrogen to ammonium, but their distribution and activity can only be quantified through sampling and experiments during research cruises. Some recent studies document small diatoms with symbionts able to fix nitrogen, a new source of biologically available nitrogen in addition to the well-known cyanobacterial species such as *Trichodesmium* or symbionts of haptophytes (UCYN-A) and diatoms (Diatom–Diazotroph Associations, or DDAs). Here, we document a very active symbiosis between small pennate diatoms such as *Mastogloia* and *Haslea* with rhizobial and cyanobacterial symbionts in waters of the Western tropical North Atlantic influenced by the Amazon River plume. We used NanoSIMS analysis of ^15^N_2_ tracer experiments to quantify high rates of nitrogen fixation in generally abundant, symbiont-bearing pennate diatoms. This newly described symbiosis may contribute a previously unquantified flux of biologically available nitrogen to oceanic systems. Pennate diatoms and their symbionts may close a key gap in our understanding of the supply of nutrients to the ocean and provide a previously unknown biological sink for carbon dioxide.

Dinitrogen (N_2_) fixation plays a key role in supplying reactive nitrogen to pelagic ecosystems, and filamentous cyanobacteria were long thought to be the only marine diazotrophs. In recent years, the application of fluorescence analysis, molecular and bioinformatic approaches, and single-cell mass spectrometry has generated increasing evidence for a significant contribution of new nitrogen by other groups including non-cyanobacterial diazotrophs [1] and newly discovered endosymbionts of haptophytes [2] and diatoms [3, 4]. Interestingly, these latter diazotrophs could represent an early stage of organellogenesis [5].

Among the enigmatic marine diazotrophs are spheroid bodies containing N_2_ fixation genes [6] in rhopalodiacean diatoms such as *Rhopalodia gibba* [7]. Similarly, Nakayama [8] found non-photosynthetic intracellular cyanobacteria in the rhopalodiacean *Epithemia turgida* [9], and N_2_ fixation has been documented in other rhopalodiaceans [4]. More recently, Tschitschko *et al*. [3] have described a rhizobial symbiont, *Candidatus Tectiglobus diatomicula*, of biraphid diatoms belonging to the genus *Haslea*. The rhizobial symbiont appears to be broadly distributed in the world ocean [3], but its host specificity and activity remain poorly characterized. Taken together, these different lines of evidence suggest an overlooked contribution of symbiosis between diatoms and multiple diazotroph lineages to nitrogen supply in the ocean.

Here, we report unexpectedly high ^15^N enrichment within pennate diatoms tentatively identified as *Mastogloia* spp. and *Haslea* spp. from ^15^N_2_—tracer experiments carried out in the northern Amazon River Plume (Figs 1 and 2, Supplementary Fig. S1, Supplementary Tables S1–S3). We could not visualize the rhizobial symbionts reported from of *Haslea* [3] and *Epithemia* [4], but many of the diatoms we observed contained symbionts with phycoerythrin fluorescence flanking the nucleus axially, in contrast to the lateral arrangement of the previously described rhizobial symbionts (Fig. 2, Supplementary  Fig.  S3). Our Nano-SIMS analyses of ^15^N in individual diatoms provide definitive evidence of direct assimilation of ^15^N_2_ by cells containing these endosymbionts (Fig. 1).

**Figure 1:**
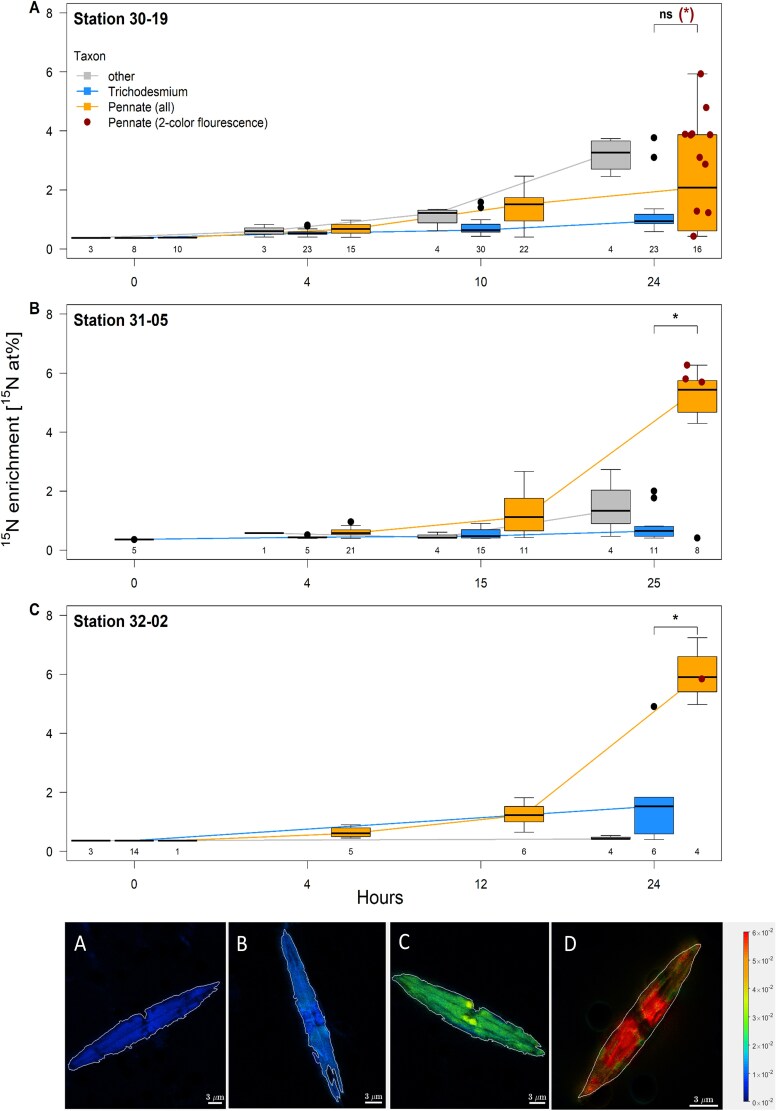
^15^N enrichment in at% in cells of Trichodesmium, pennate diatoms, and other diazotrophs such as Rhizosolenia-Richelia, Chaetoceros over the incubation time of up to 25 h from three stations (A, station 30-19; B, station 31-05; and C, station 32-02) in the Amazon river plume. Boxplots display the median and interquartile range with whiskers extending to the minimum and maximum, excluding outliers. Outliers are shown as dots and defined as values more than 1.5 times the interquartile range removed from either the lower or upper quartile. Numbers below the boxplot indicate the number of cells measured. Significant differences in ^15^N enrichment after 24–25 h between *Trichodesmium* and all analyzed pennate diatoms are indicated by asterisks, ns means not significant. Depth was not considered as a factor since it did not affect ^15^N enrichment (Wilcoxon rank sum test, *P* > .05). At the end of the incubation, the ^15^N enrichment of pennate diatoms with two color fluorescence is shown as red points and the significance of the difference with resp. to Trichodesmium enrichment is indicated for station 30-19 in parentheses. Typical examples of the enrichment of pennates are shown in the panels (D–G) below for the different time points 0 (D), 4 h (E), 10–14 h (F), and 24–25 h (G). The scale bar illustrates enrichments with ^15^N from zero to 6 × 10^−2^ ‰ (cold to warm colors from left to right).

**Figure 2:**
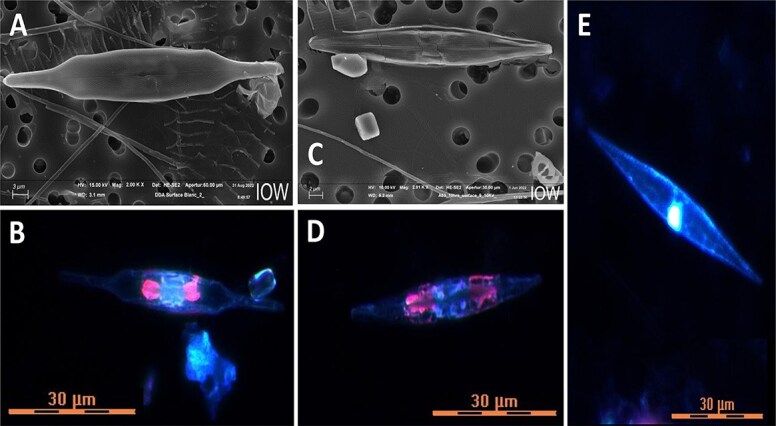
Examples of pennate diatoms showing putative *Mastogloia* sp. (A, B) and *Haslea* sp. (C, D) cells with two-color fluorescence. Panels (A and C) show general morphology, while panels (B and D) show fluorescence under trichromatic excitation (see Supplementary Fig. S3 for details). In contrast, panel (E) shows a diatom lacking putative symbionts and exhibiting monochromatic fluorescence.

Although pennate diatoms are known to acquire reactive nitrogen from sympatric, free-living diazotrophs [4], this pathway cannot generate ^15^N enrichments greater than those in the free-living diazotrophs. In all, we identified 108 pennate diatoms that became enriched in ^15^N, of which 56 contained two-color fluorescent inclusions indicative of cyanobacteria (Supplementary Table S4). At the end of our incubations, many pennate diatoms had significantly higher ^15^N enrichments than *Trichodesmium* and other Diatom-Diazotroph Associations previously known to supply reactive nitrogen to the Amazon River Plume [9–11] (Fig. 1, Supplementary Tables S4–S6).

The high ^15^N enrichment of the pennate diatoms with fluorescent symbionts implies that they were not acquiring ^15^N secondarily from other diazotrophs via a recycling pathway. As a quantitative test, we estimated that the maximum possible enrichment of ^15^N in pennates via release of labeled ^15^N-NH_4_^+^ by other active diazotrophs in the surrounding water is ~1.15 at% ^15^N, a third of the median enrichment of 3.89 at% (*n* = 28) we found in pennates after 24 h of incubation (SI-Methods). The ^15^N enrichment of pennate diatoms with symbionts increased through our 24 h incubations to as high as 7.24 at% ^15^N (Fig. 1, Supplementary Table S4), while the significantly lower ^15^N enrichments of pennates without symbionts are consistent with acquisition of ^15^N via a recycling pathway (Dunn’s test, *z* = 4.12, *P* < .001).

Because these findings were serendipitous, we lack molecular analyses allowing us to unambiguously resolve the identity of these fluorescent endosymbionts. However, we collected samples for metatranscriptomics and interrogated them for community-level signals of *nifH* gene transcription as well as diatom taxonomy to explore the potential identity of both symbionts and hosts. For taxonomic assessment, Kaiju, a protein-level classification tool [12], could classify between 13.6% and 45.7% of metatranscriptomic reads (Supplementary Table S3) revealing relatively similar diatom communities across the sampled region with site-by-site variability (Supplementary Fig. S4).

Among the diatoms, transcripts attributed to *Mastogloia* spp. occurred at all stations with the highest relative abundance at station 29-04 (Supplementary Fig. S4B), while transcripts attributed to *Haslea* spp. were highest at station 31-05 (Supplementary Fig. S4B), suggesting broad presence but relatively low contribution of these pennates to the diatom community. Pennate diatoms of these genera benefit from elevated silicate concentrations e.g. in the river plumes like that of the Amazon (Supplementary Table S1) [11, 13] and may be observed elsewhere.

Although we could not identify the symbionts of our diatoms unambiguously, an evaluation of putative *nifH* gene expression within the assemblage using a combination of blast searches versus curated and public databases revealed the presence of the rhizobial symbiont, *Candidatus Tectiglobus diatomicola*, in our samples despite its absence in our microscopic observations (Supplementary Fig. S5). Overall, the highest *nifH* transcript proportion was observed at Station 28-01 for transcripts attributed to *Trichodesmium* (3.84 TPM), while transcripts belonging to the recently identified rhizobial symbiont (*Candidatus Tectiglobus diatomicola*, [3]) showed the highest expression at Station 29-04 (1.87 TPM; Supplementary Fig. S5). Our samples also contained transcripts attributed to *Richelia*, *Crocosphaera*, UCYN-A, and an unclassified cyanobacterium (Supplementary Fig. S5). Although we cannot yet identify the inclusions associated with high rates of assimilation of ^15^N by pennate diatoms, they have morphological and fluorescence characteristics of cyanobacteria. Our methods did not allow us to visualize the previously described rhizobial symbionts of *Haslea*, but our molecular data show them to be present (Fig. S5). Our observations thus provide strong evidence that pennate diatoms may form symbiotic associations with both rhizobia and currently unidentified cyanobacteria.

In summary, multiple broadly distributed diatom genera may be associated with rhizobial and/or cyanobacterial symbionts, but the small size of the pennate diatom host and the challenge of characterizing Diatom–Rhizobial Associations (DRAs) and some Diatom–Cyanobacterial Associations (DCAs) by light or fluorescence microscopy has led to them being largely overlooked. Pennate diatoms are abundant and broadly distributed but whether they play an important role in supplying new nitrogen to tropical waters of the North Atlantic cannot yet be assessed. Further field studies are needed to explore the possibility that DRAs and DCAs with pennate diatom hosts are active in other ocean basins, and to quantify their overall contribution to the oceanic nitrogen budget.

## Supplementary Material

Panthalil_Vogts_ISME_Comm_SI_final_ycaf190

## Data Availability

Fastq files have been deposited at the National Center for Biotechnology Information (NCBI) Sequence Read Archive (SRA) https://www.ncbi.nlm.nih.gov/sra under accession number PRJNA1226575. METEOR cruise M174 [Dataset]. Hydrographic properties of water masses in the Amazonas River plume obtained in April/May 2021 by CTD measurements during RV METEOR cruise M174 PANGAEA, https://doi.org/10.1594/PANGAEA.942346. A, NanoSIMS data published as supplementary tables with this article.
