# Supplementary material for: Novel pennate diatom symbionts support high N2 fixation rates
Source: ISME Commun. 2025 Oct 24;5(1):ycaf190. doi: 10.1093/ismeco/ycaf190 (PMC12619531; doi:10.1093/ismeco/ycaf190)
Supplement: Panthalil_Vogts_ISME_Comm_SI_final_ycaf190 [file panthalil_vogts_isme_comm_si_final_ycaf190.docx]

**Supplementary Information**

**Novel pennate diatom symbionts support high N_2_ fixation rates**

Bhavya S. Panthalil^1,$,*^, Angela Vogts^1,*^, Mar Benavides^2,3,4^, Matthew J. Harke^5^, Christiane Hassenrück^1^, Ajit Subramaniam^6^, Joseph P. Montoya^7^, Maren Voss^1,*^

^1^Leibniz Institute for Baltic Sea Research Warnemünde, Rostock, 18119, Germany

^$^Indian Institute of Science Education and Research; IISER, Thiruvananthapuram, Kerala, 695551, India

^2^National Oceanography Centre, European Way, Southampton, SO14 3ZH, United Kingdom

^3^Aix Marseille Univ, Université de Toulon, CNRS, IRD, MIO UM 110, 13288, Marseille, France

^4^Turing Centre for Living Systems, Aix-Marseille University, 13009 Marseille, France

^5^Gloucester Marine Genomics Institute, Gloucester, Massachusetts, 01930, United States

^6^Lamont-Doherty Earth Observatory, Palisades, New York 10964, United States

^7^Georgia Institute of Technology, Atlanta, Georgia 30332, United States

*Shared first authorship

Corresponding author: Maren Voss [maren.voss@io-warnemuende.de](mailto:maren.voss@io-warnemuende.de)

**The supplementary information includes the following:**

Supplementary Methods

Figures S1-S5

Tables S1-S6

References

**Supplementary Methods**

*Sampling and incubations*

We collected samples in the Amazon River Plume region during cruise M174 aboard F/S Meteor between May 9^th^ and May 13^th^ 2021 (Table S1). This cruise took place during the high outflow season of the Amazon River, which generates a plume that extends from the river mouth on the equator to >15°N. In the Plume, low density river-derived water flows above higher density marine water masses. We sampled the water column between 14.1 and 14.8 °N (Fig. S1, Table S1) using a Seabird Electronics SBE-911plus (SN-0603) Conductivity-Temperature-Depth (CTD)-rosette system equipped with 20 Niskin bottles of 10 l volume. Vertical profiles of temperature, salinity, dissolved oxygen, turbidity, and fluorescence were measured with sensors of CTD and SBE43 dissolved oxygen sensor. Samples from the surface (ca. 4 m) and at the mixed layer (transition zone to lower marine water masses, 15-20 m) were sampled. The mixed layer depth was determined as the maximum in buoyancy frequency.

Dissolved nutrient concentrations (nitrate-NO_3_^-^, nitrite-NO_2_^-^, ammonium-NH_4_^+^, phosphate-PO4_3_^-^, and silicateSiO_3_^2-^) were measured from samples collected from the same CTD casts and filtered (0.2 µm) immediately after collection. Nutrients were measured colorimetrically according to [1] using a Seal Analytical QuAAtro automated constant flow analyser with a precision of 0.3 μM (Si[OH]_4_), 0.02 μM (NO_3_^−^), and 0.01 μM (PO_4_^3-^).

For the metatranscriptome samples four litres of water was collected using Niskin bottles, usually from the surface, as soon as the CTD rosette came aboard and filtered through 0.2 µm pore Sterevix cartridge filters. Filtration was terminated after 30 minutes and the volume filtered was noted. The Sterivex cartridges were capped and stored in liquid N_2_ immediately, transported to the lab in a dry cryoshipper and then stored at -80ºC till shipped in cryoshipper to the Sequencing Facility.

Sample stations for incubation experiments were selected based on shipboard microscopy analyses: Surface seawater (4 m) samples were collected and concentrated with 10 µm mesh, a subsample was then transferred to a microscope slide and screened for the presence of diazotrophs and diatom-diazotroph association using an epi-fluorescence microscope (Zeiss Axion ERc 5c) with an excitation wavelength of 470nm.

Samples for N_2_ fixation measurements were transferred directly from Niskin bottles of the (CTD)-rosette to 500 ml acid-washed polycarbonate bottles (Nalgene, Thermo Fisher Scientific, USA). For each time-point, duplicate bottles were filled bubble free and closed tightly with septum caps. Each bottle was amended with 2 ml ^15^N_2_ gas (99% ^15^N, Cambridge isotope laboratory, USA) resulting in a maximum enrichment of roughly 45% at^15^N_2_. Although the gas from Cambridge Scientific has not been described as contaminated to date, we have checked the N_2_ gas bottles for ammonia contamination [2] before use by injecting a small subsample of the gas (approx. 10 ml) into acidified MilliQ water (pH 1-2) which would immediately dissolve gaseous NH_3_ and protonate it to NH_4_^+^. A NH_4_^+^ concentration determination [1] was then carried out, which was below detection limit. Since even low ammonia concentrations in the N_2_ gas bottles may bias the δ^15^N data of an incubation we furthermore injected a subsample of the N_2_ gas into a lightly acidic solution with a standardized background NH_4_^+^ concentration of 5µM to bring the detection limit into a well measurable range. By means of the diffusion method [3, 4] the solution was then analysed for δ^15^N-NH_4_^+^ signatures. We found a value of -2.23 ± 0.5‰ (n=6) in the δ^15^N-NH_4_^+^ which corresponds to the background δ^15^N-NH_4_^+^ value confirming no contamination in the tracer N_2_ gas bottle.

We used the bubble method, as opposed to the enriched water method, to minimize the physical disturbance of the sample as described in White [5]. However, we recognize the potential to underestimate rates over short time intervals [6], thus our experiments provide a lower bound on actual nitrogen fixation rates in situ. Incubations were carried out in deck incubators supplied with a continuous flow of surface seawater at ambient temperature. Bottles containing water from the bottom of the plume were covered with neutral density screening to simulate in situ light conditions (25% surface irradiance). We terminated our incubations by gentle pressure filtration onto 25 mm Millipore membrane filters with a pore size of 3 μm after 4, 10-15, and 24-25 h of incubation, respectively. Filters were fixed with formaldehyde solution, air-dried and stored frozen at -20°C until analysis. We also collected time zero (t_0_) samples to measure the initial isotopic composition of organisms by filtering water immediately after collection without ^15^N_2_ tracer addition. Samples were stored in the dark at -20°C for later analysis in the lab.

*Microscopy*

Among the specimens with a fluorescence emission similar to that of *Trichodesmium*, we found several pennate diatoms as well as *Chaetoceros-Richelia* and *Risozolenia-Richelia*-associations. In addition, two types of small araphid pennates showed an additional fluorescence emission different than that of chlorophyll in proximity to the chloroplast, indicative of the presence of unknown symbionts/organelles located near the nucleus. This fluorescence does not resemble any observations of published symbiosis between pennate diatoms and bacteria ([7] [8]) (Fig. S3). Detailed laser dissecting microscope image analysis on selected specimens was performed with excitation illumination at 400±15, 490±15, and 570±25 nm, and associated emissions at 460±25 (blue), 535±35 (green), 630±60nm (red) and bright field, respectively. The fluorescent structures were 5-8 µm in diameter, smaller than the common cyanobacterial symbiont, *Richelia* spp. (ca. 30 µm and in chains) but larger than the spheroid bodies previously reported in rhopalodiacian diatoms (ca. 3 µm in diameter) [9] as well as the previously described rhizobial symbionts, which are 1-2µm in diameter [8]. The fluorescent structures were arranged longitudinally on either side of the nucleus, in contrast to the rhizobial symbiont recently found in *Haslea*, which is located lateral to the nucleus [8] or symbionts in *Epithemia [7]*. Since the fluorescence patterns and structures we observed differ from the results published to date (Fig. S4), we believe we have found additional diatom symbionts that are capable of fixing N_2_.

A few specimens were inspected under the scanning electron microscope (Merlin Compact VP Scanning Electron Microscope, Zeiss Germany at the Leibniz Institute for Baltic Sea Research Warnemünde). for the SEM analysis we used 1 centimeter diameter sample filter pasted on a metallic stub with laser marked specimens and coated with iridium. This enabled us to unambiguously identify the morphological features of *Mastogloia rostrata* and *Halsea spp*. in selected specimens by comparison with the images of [10]https://diatoms.org/morphology/symmetric_biraphid. Specifically, the valves of *Mastogloia rostrata* are elliptic with capitate ends and the axial area is very narrow and encloses the nearly straight raphe fissures (Figure S2). The proximal raphe ends flex slightly in the same direction at the small central area and their distal ends terminate at the valve apices as small lacriform expansions.

The microscopic identification of *Haslea* was performed based on SEM imaging (Figure 2B) and comparison with information from (Simonsen) [11] and the webpage diatoms.org. The key characters we used were the lanceolate valves with acute apices, the well-defined longitudinal striae on the valves, and the appearance of the transapical striae, which appear modified with damaged longitudinal ribs (Fig. 2B) as in the study of [8]. The raphe endings were closely spaced (information given in [11].

We were not able to carry out a detailed morphological examination of every specimen analysed, usually because of cell orientation and/or the presence of other material or cells obscuring parts of the surface. However, for at least 85% of the enriched pennates frustule shape and size resembled those of *Mastogloia spp.* and *Haslea spp*.

Both species are reported to be ubiquitous in the world’s oceans [12-14] as did the study of Tschitschko. The occurrence in tropical waters of the Atlantic and pacific ocean and significance of small diatoms, in particular of the transport towards depth, has been described by Scharek et al. [15].

*NanoSIMS analysis*

For NanoSIMS analysis, samples were allowed to come to room temperature in a darkened extractor hood for 4 h. Circular subsamples of 1 cm diameter were excised from the filter and attached to a metal stub with conductive tape. We used a Leica Laser Micro Dissection (LMD) microscope at 10x magnification to identify *Trichodesmium*, pennate diatoms, and other organisms based on their fluorescence properties. All images were analysed based on 63x magnification. Spots of interest were marked with the laser beam.

The samples were coated with ca. 30 nm gold with a Cressington 108auto sputter coater (Watford, United Kingdom). At marked positions, SIMS imaging was performed using a NanoSIMS 50L instrument (Cameca, Paris, France) at the Leibniz-Institute for Baltic Sea Research Warnemünde (IOW). A ^133^Cs^+^ primary ion beam was used to erode and ionize atoms of the sample. Images of secondary electrons and ^12^C^14^N^-^, ^12^C^15^N^-^ ions were recorded simultaneously using mass detectors equipped with electron multipliers. The mass resolving power was adjusted to be sufficient to suppress interference at all masses allowing us to separate ^12^C^15^N^-^ from interfering ions such as ^13^C^14^N.

Prior to the analysis, sample areas measuring 50×50 µm were sputtered for 2 min at 600 pA to erode the gold, clean the surface and reach the steady state of secondary ion formation. For positions with pennates additional 5 min sputter intervals were added to erode the silicate and expose the organic matter inside. After each interval the sample spot was inspected by real time imaging to decide if the organic matter signal was sufficient. 1-10 sputter intervals were employed with most pennates requiring 3-4 sputter intervals to remove enough silicate to expose the cell contents. The primary ion beam current during the subsequent analysis was 1 pA; the scanning parameters were 512×512 pixels for areas of 25x25 to 45×45 µm, with a dwell time of 250 µs per pixel. From each sample, 60 planes were analysed.

Data analysis was performed with the Look@NanoSIMS software [16]. The planes were checked for inconsistencies, drift corrected and accumulated. For regions of interest (ROIs) selected based on mass ^12^C^14^N^-^. The ^15^N at % was calculated as ^12^C^15^N^-^/(^12^C^14^N^-^+^12^C^15^N^-^) based on the ion counts averaged over the ROIs. NanoSIMS derived ^15^N ratios were corrected for instrumental fractionation. Factor = 0.09782, based on the nominal value for *Trichodesmium* (0.3661, [17].

To prevent a bias by the different abundance of non-enriching species among the large multi-species groups of pennates and others, we only took into account specimens with enrichment higher than the average value for t0+3ơ (higher than 0.4% ^15^N).

*Potential labeling of pennate diatoms by ^15^NH_4_^+^ released by other diazotrophs*

In order to verify that the pennate diatoms are able to fix N_2_^-^ independently of other, well-known diazotrophs, we estimated the potential rate of transfer of ^15^N from *Trichodesmium* to the diatoms based on the following assumptions: A) *Trichodesmium* fixes N_2_ at a rate of 0.41 to 0.58 (nmol l^-1^ h^-1^ , Umbricht et al. pers. comm), B) filaments release 50% of the nitrogen they fix as NH_4_^+^ [18], C) there is no other NH_4_^+^ available in the water column; and D) the ^15^N at% of released NH_4_^+^ is equivalent to the ^15^N enrichment of the N_2_ pool, the upper limit of the potential ^15^N enrichment of the ammonium available for other phytoplankton to assimilate. We further assumed that the pennate diatoms would assimilate all of the released NH_4_^+^ during the course of a 24-hour incubation period. With these assumptions, we estimated that the final ^15^N at% of pennate diatoms would be 1.15 ± 0.16 (n=4). Enrichments of the pennates measured with NanoSIMS were much higher, with a median enrichment of 3.89 ^15^N at% and a maximum of 7.2 ^15^N at%. This 3- to 6- fold difference in ^15^N enrichment is strong evidence that the pennates have their own N_2_-fixing mechanism and are not dependent on *Trichodesmium* or other diazotrophs to fix nitrogen. Although the pennates may also consume the released fixed N in parallel to their internal nitrogen fixation, this external source makes at most a minor contribution to their assimilation of ^15^N_2_^-^ tracer.

*Statistical analysis*

At the end of the incubation, we compared the ^15^N enrichment of cells of different taxonomic groups (*Trichodesmium*, pennate diatoms) and different sampling using the non-parametric Scheirer-Ray-Hare test, followed by a post-hoc Dunn’s test for pairwise multiple comparisons with a Bonferroni p-value correction. The non-parametric approach was chosen since the enrichment data were not normally distributed. ^15^N enrichment did not differ between water depths, so this factor was not considered in the statistical analysis (Wilcoxon rank sum test, p > 0.05). Furthermore, the difference between ^15^N enrichment in *Trichodesmium* cells and only those pennate diatoms which displayed two-color fluorescent signal was compared with a Wilcoxon rank sum test at station 30.

*Molecular analyses and bioinformatics*

Total nucleic acids were extracted from Sterivex filters collected at the stations given in table S1 using a Macherey-Nagel NucleoMag^®^ DNA/RNA Water Kit following manufacturer’s instructions for Sterivex™ filter units with the following modifications. For lysis, 1 mL of Lysis Buffer MC1 was added to the Sterivex filter unit and after agitation and incubation, a bead beating step was addied by transferring lysate to a Macherey-Nagel Bead Tube Type A, and vortexing for 4 min. Resulting lysate was then split in two and individually processed for DNA and RNA separately. Binding of nucleic acids to NucleoMag B-Beads was conducted in 1.5 mL microcentrifuge tubes with final elution volume of 50 µL. Samples for RNA isolation were DNase treated using a Qiagen DNase Max^®^ Kit, following manufacturer’s instructions. The quantity and quality of resulting total RNA was assessed with a Qubit and Fragment Analyzer with a high sensitivity RNA kit, and stored at -80°C until library preparation and sequencing.

Total RNA samples (250 ng of Qubit quantified total RNA input) were prepared for metatranscriptomic sequencing at the University of Connecticut Center for Genome Innovation (<https://cgi.uconn.edu/>) using the Illumina Stranded Total RNA Prep, Ligation with Ribo-Zero Plus library preparation kit (Illumina, San Diego, CA) following the manufacturer’s protocol. Libraries were validated for length and adapter dimer removal using the Agilent Tape Station 4200 D1000 High Sensitivity assay (Agilent Technologies, Santa Clara, CA, USA), then quantified and normalized using the dsDNA High Sensitivity Assay for Qubit 3.0 (Life Technologies, Carlsbad, CA, USA). Sample libraries were then prepared for Illumina sequencing on the NovaSeq 6000 by denaturing and diluting the libraries per manufacturer’s protocol (Illumina, San Diego, CA, USA). All samples were combined into one sequencing pool, proportioned according to expected number of reads, and run as one sample pool using a S4 200 cycle v1.5 flow cell targeting 60M 100bp paired end reads per sample.

Reads were assessed for quality using FastQC (<https://www.bioinformatics.babraham.ac.uk/projects/fastqc/>) and trimmed using Trimmomatic (SLIDINGWINDOW:5:20 HEADCROP:10 MINLEN:40 and removing adapters). Trimmed reads were cleaned of rRNA contamination using SortMeRNA v4.3.6 [19] and assembled using rnaSPAdes v3.15.5 [20]. Transcript abundances were estimated with RSEM v1.3.3 [21] and putative annotations were assigned using Prokka v1.14.5 [22]. Eukaryotic taxonomy was assigned to non-assembled reads using Kaiju [23] with the prebuilt nr_euk reference database. Resulting count tables were separated by diatom taxa, square-root transformed, and assessed for community differences among stations using Bray-Curtis dissimilarity calculated with the ‘vegdist’ function within the R package ‘vegan’ (version 2.6-6.1). In addition, *nifH* taxonomy was queried in two ways. First, transcripts identified as *nifH* through Prokka annotation were blasted (blastX) against the non-redundant protein sequence (nr) database (NCBI) with the best taxonomy hit assigned. Secondly, assembled transcripts were also blasted (blastN) against a curated *nifH* reference database [24]. Blast results from both methods were evaluated manually to remove any hits with length <100 and the best hit being retained (lowest e-value, lowest mismatch, and highest percent identity). Fastq files have been deposited at the National Center for Biotechnology Information (NCBI) Sequence Read Archive (SRA) https://www.ncbi.nlm.nih.gov/sra under accession number [PRJNA1226575](https://www.ncbi.nlm.nih.gov/bioproject/PRJNA1226575).

**Supplementary Figures**


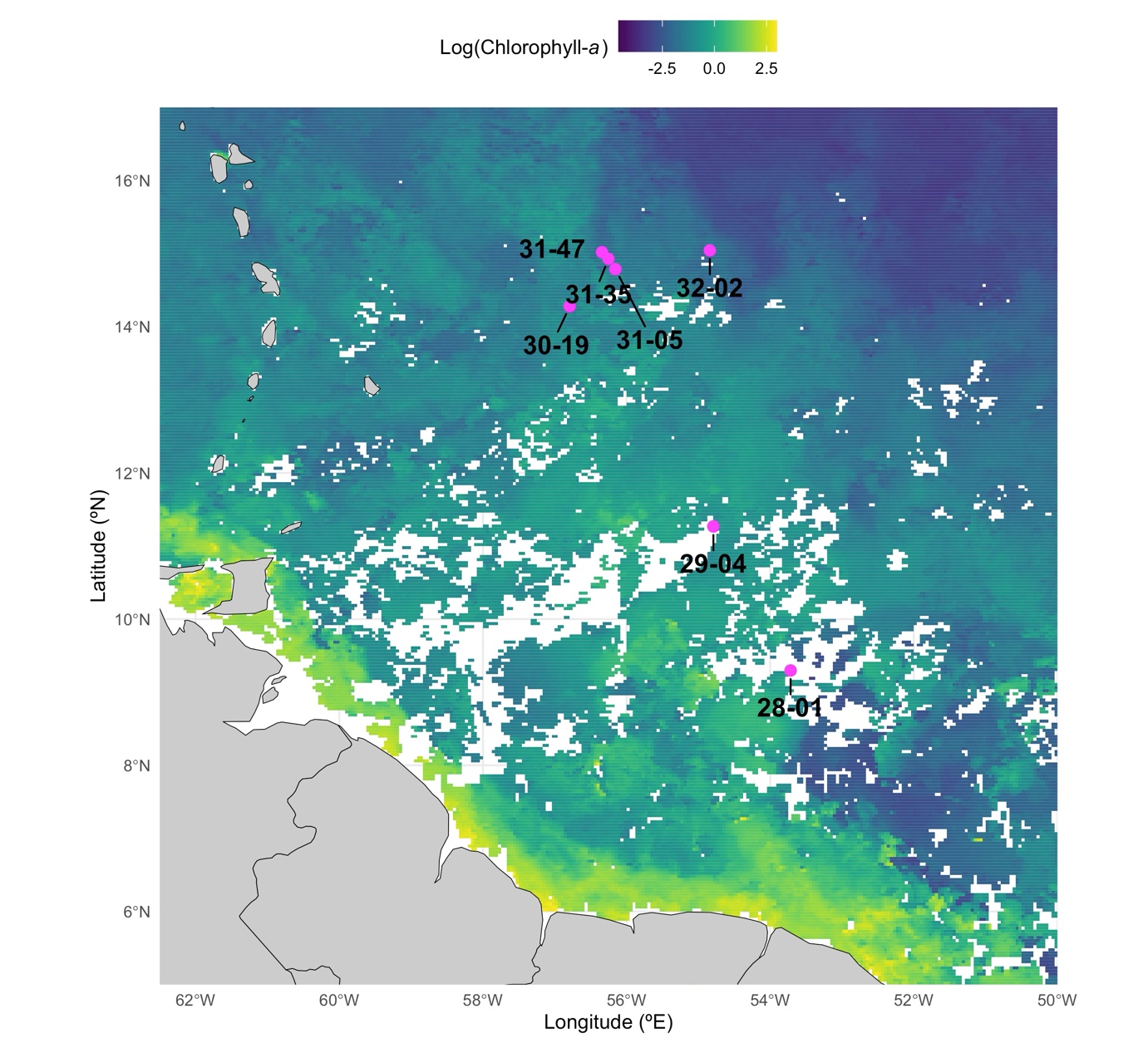


Fig. S1: Stations sampled during the M174 cruise overlaid on a Chlorophyll-*a* (mg m^-3^) composite of L3M 4 km product retrieved from the Copernicus marine service for the cruise period (May 2021).


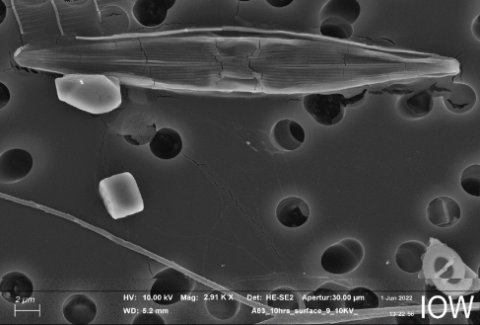

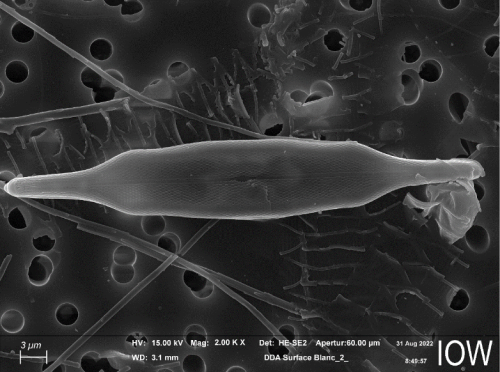


A

B

1

1

2

3

3

4

Fig. S2: the same images as in Fig. 2. *Mastogloia rostrata* with elliptic valves with capitate ends (1), the axial area encloses the nearly straight raphe fissures (2). The proximal raphe and its end and their distal ends as small lacriform expansions cannot be seen in this image (A). *Haslea* has lanceolate valves with acute apices (3), the well-defined longitudinal striae on the valves with fine poroids (4).

(A)


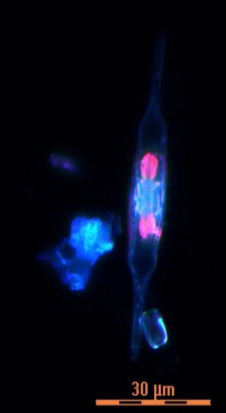

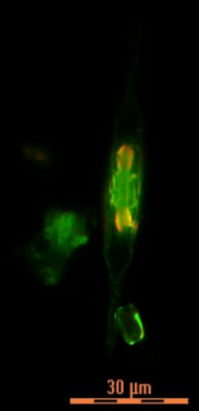

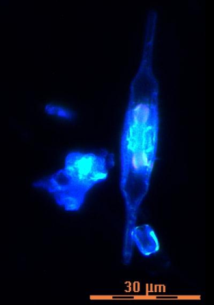

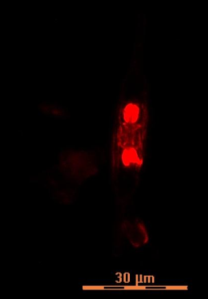


**A**

**B**

**C**

**D**

Fig. S3: Fluorescence images taken with a Laser MicroDissection microscope of the pennate diatom shown in Fig. S2 (A) combined blue, green, red light image; with the symbiont in fluorescing pink, (B) UV excitation, blue emission; (C) green excitation, yellow emission of phycoerythrin in the cyanobacterial symbiont; and (D) yellow excitation, red emission of chlorophyll in cyanobacterial symbiont.


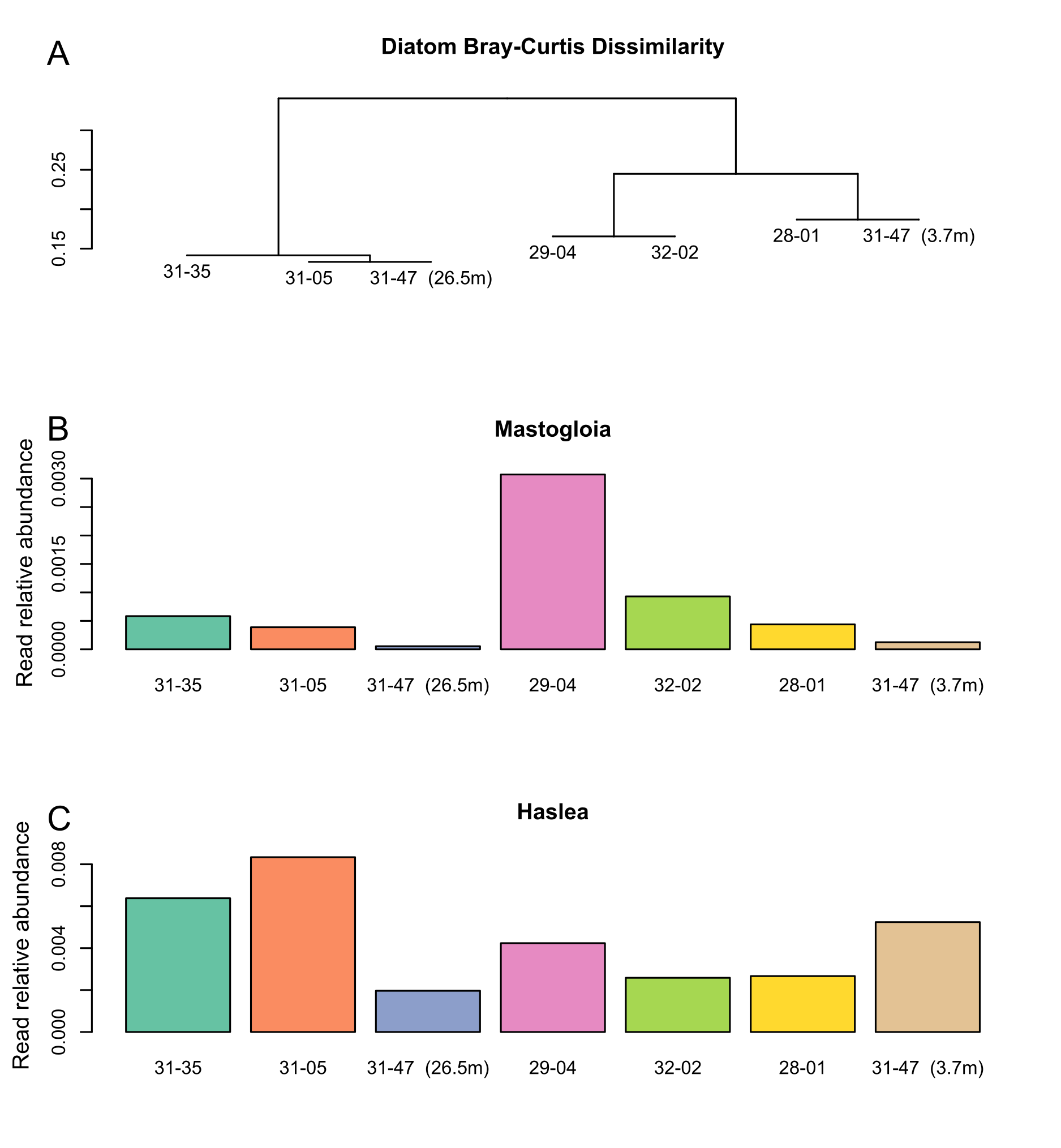


Fig. S4. A) The Bray-Curtis dissimilarity of reads attributed to diatoms across the sampled stations, B) the relative read abundance of reads attributed to *Mastogloia* sp., and C) the relative read abundance of reads attributed to *Haslea spp*.


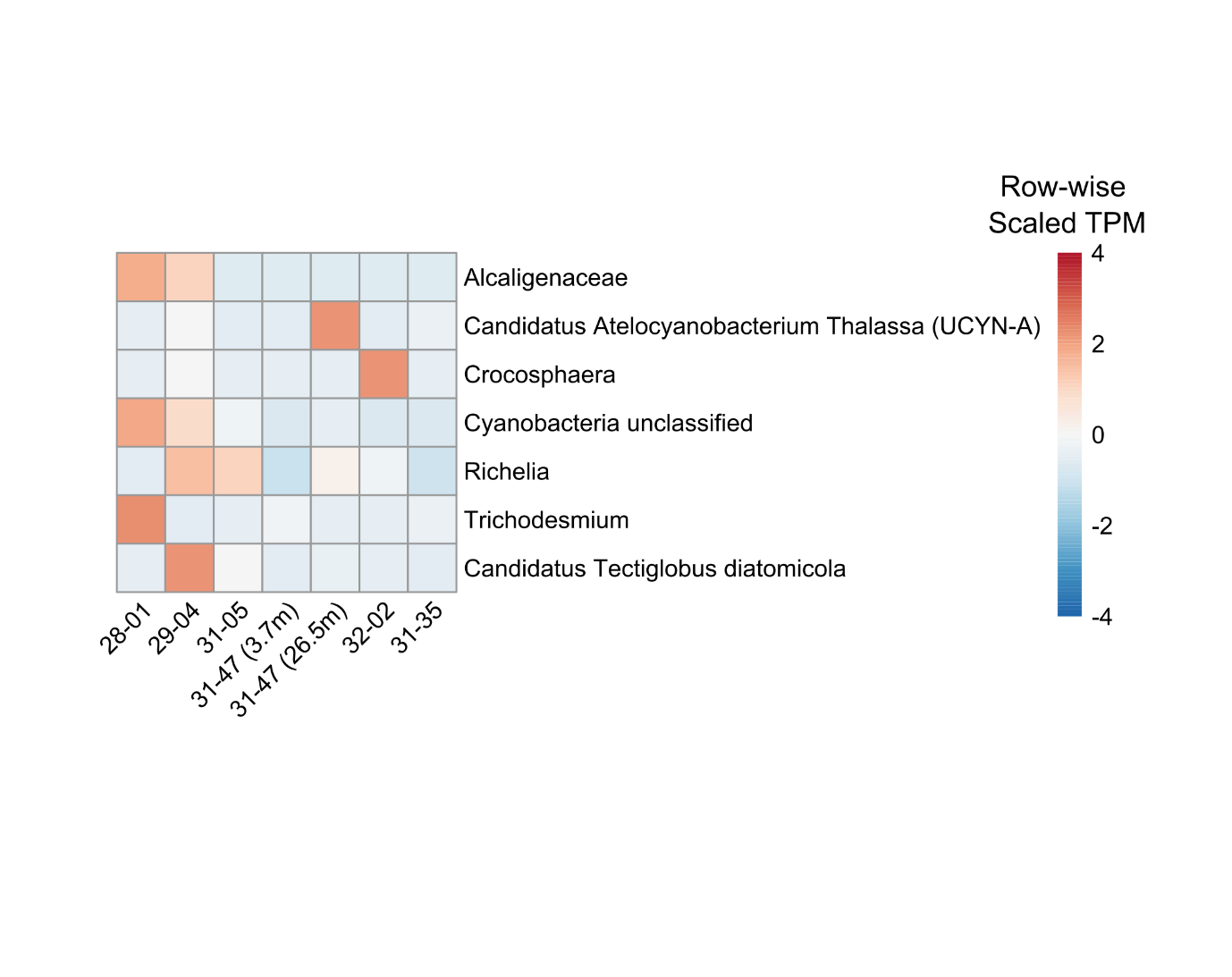


Fig. S5: Heatmap displaying *nifH* gene expression in row-wise scaling of transcript per million (TPM) values for putative diazotrophs at each station. Symbionts are *Candidatus Atelocyanobacterium Thalassa* (with a haptophyte), Richelia (with diatoms) and *Candidatus Tectiglobus diatomicola* (with a diatom).

**Supplementary Tables**

Table S1: Sampling Locations for metatransciptome data and incubations for fluorescence microscopy and NanoSIMS analyses and nutrient concentrations in respective sampling depths.

| Date | Station Number | CTD Number | Sample Collected | Longitude (°E) | Latitude (°N) | Sampling time  (UTC) | Incubation start (UTC) | Depth (m) | Temp. (°C) | Salinity | PO_4_^3-^  (µmol L^-1^) | NO_3_^-^  (µmol L^-1^) | Silica (µmol L^-1^) |
| --- | --- | --- | --- | --- | --- | --- | --- | --- | --- | --- | --- | --- | --- |
| May 07 2021 | 28-01 | 81 | Genomics | -53.716 | 9.297 | 18:45 | N/A | 2.4 | 27.83 | 32.38 | 0.06 | 0.07 | 7.37 |
| May 08 2021 | 29-04 | 85 | Genomics | -54.791 | 11.267 | 14:00 | N/A | 2.6 | 27.52 | 33.90 | 0.05 | 0.08 | 4.20 |
| May 09 2021 | 30-19 | 90 | NanoSIMS NanoSIMS | -56.791 | 14.277 | 12:00 | 13:10 | 3.2 20.9 | 27.37 27.26 | 31.20 35.09 | 0.04 0.05 | 0.10 0.34 | 6.68 5.18 |
| May 11 2021 | 31-05 | 98 | NanoSIMS, Genomics NanoSIMS | -56.154 | 14.785 | 16:30 | 17:20 | 2.8 15.8 | 26.57 26.59 | 33.64 35.11 | 0.02 0.01 | 0.03 0 | 3.38 2.40 |
| May 12 2021 | 31-35 | 104 | Genomics | -56.252 | 14.927 | 14:09 | N/A | 2.7 | 26.65 | 33.80 | 0.04 | 0.59 | 3.15 |
| May 13 2021 | 31-47 | 107 | Genomics Genomics | -56.341 | 15.018 | 00:02 | N/A | 3.7  26.5 | 26.67 26.52 | 33.55 36.26 | 0.02 0.02 | 0.06 0.03 | 3.00 1.48 |
| May 13 2021 | 32-02 | 112 | NanoSIMS, Genomics | -54.840 | 15.041 | 19:43 | 20:45 | 3.5 | 26.91 | 34.49 | 0.04 | 0.61 | 2.95 |

Table S2: Result of the Scheirer-Ray-Hare test for the 2-way analysis of the differences in ^15^N enrichment between taxonomic groups and sampling stations.

|  | Degrees of freedom | Test statistic (H) | P-value |
| --- | --- | --- | --- |
| Taxon | 1 | 13.372 | <0.001 |
| Station | 2 | 3.918 | 0.141 |
| Interaction | 2 | 8.158 | 0.017 |

Table S3: Results of Dunn’s post-hoc tests to compare pairwise differences between taxonomic groups per station and between stations per taxonomic group.

|  | Group | Comparison | | Number of observations | | Statistic (z) | P |
| --- | --- | --- | --- | --- | --- | --- | --- |
| Station | | Group 1 | Group 2 | Group 1 | Group 2 |  |  |
|  | Station 30-19^a^ | *Trichodesmium* | Pennate diatoms | 23 | 16 | 1.20 | 0.691 |
|  |  | *Trichodesmium* | Pennates without 2-color | 23 | 11 | -2.31 | 0.063 |
|  |  | *Trichodesmium* | Pennates with 2-color | 23 | 5 | 2.96 | **0.009** |
|  |  | Pennates without 2-color | Pennates with 2-color | 11 | 5 | 4.12 | **<0.001** |
|  | Station 31-05 | *Trichodesmium* | Pennate diatoms | 11 | 8 | 2.81 | **0.015** |
|  | Station 32-02 | *Trichodesmium* | Pennate diatoms | 6 | 4 | 2.56 | **0.032** |
| Taxon | |  |  |  |  |  |  |
|  | *Trichodesmium* | Station 30-19 | Station 31-05 | 23 | 11 | -2.20 | 0.167 |
|  | *Trichodesmium* | Station 30-19 | Station 32-02 | 23 | 6 | 0.49 | 1.000 |
|  | *Trichodesmium* | Station 31-05 | Station 32-02 | 11 | 6 | 2.03 | 0.252 |
|  | Pennate diatoms | Station 30-19 | Station 31-05 | 16 | 8 | 2.40 | 0.097 |
|  | Pennate diatoms | Station 30-19 | Station 32-02 | 16 | 4 | 3.03 | **0.015** |
|  | Pennate diatoms | Station 31-05 | Station 32-02 | 8 | 4 | 1.07 | 1.000 |

^a^ Kruskal Wallis test between *Trichodesmium*, small pennate diatoms with and without two-color fluorescence (2-color): X^2^ = 18.424, df = 2, p < 0.001. The results of Dunn’s post-hoc test are shown as part of the table.

Table S4: Table with enrichments from NanoSIMS analysis for pennates with elliptic to lanceolate valves

| Station | Depth | Icubation time | ^15^N at % | 2 colour fluorescence |
| --- | --- | --- | --- | --- |
| 30-19 | 3.2 | 0 | 0.37 | yes |
| 30-19 | 3.2 | 0 | 0.39 | yes |
| 30-19 | 3.2 | 0 | 0.39 | yes |
| 30-19 | 3.2 | 0 | 0.38 | yes |
| 30-19 | 3.2 | 0 | 0.37 | yes |
| 30-19 | 3.2 | 0 | 0.37 | yes |
| 30-19 | 3.2 | 0 | 0.38 | yes |
| 30-19 | 3.2 | 0 | 0.39 | yes |
| 30-19 | 3.2 | 0 | 0.38 | yes |
| 30-19 | 3.2 | 0 | 0.39 | yes |
| 30-19 | 3.2 | 4 | 0.43 | no |
| 30-19 | 3.2 | 4 | 0.68 | no |
| 30-19 | 3.2 | 4 | 0.53 | yes |
| 30-19 | 3.2 | 4 | 0.56 | yes |
| 30-19 | 3.2 | 4 | 0.74 | yes |
| 30-19 | 3.2 | 4 | 0.97 | yes |
| 30-19 | 3.2 | 10 | 0.40 | no |
| 30-19 | 3.2 | 10 | 0.41 | no |
| 30-19 | 3.2 | 10 | 1.35 | no |
| 30-19 | 3.2 | 10 | 1.48 | no |
| 30-19 | 3.2 | 10 | 1.54 | no |
| 30-19 | 3.2 | 10 | 1.74 | no |
| 30-19 | 3.2 | 10 | 2.04 | no |
| 30-19 | 3.2 | 10 | 2.31 | no |
| 30-19 | 3.2 | 10 | 2.47 | no |
| 30-19 | 3.2 | 10 | 0.65 | yes |
| 30-19 | 3.2 | 10 | 0.79 | yes |
| 30-19 | 3.2 | 10 | 0.95 | yes |
| 30-19 | 3.2 | 10 | 0.96 | yes |
| 30-19 | 3.2 | 10 | 1.26 | yes |
| 30-19 | 3.2 | 10 | 1.57 | yes |
| 30-19 | 3.2 | 10 | 1.62 | yes |
| 30-19 | 3.2 | 10 | 1.70 | yes |
| 30-19 | 3.2 | 10 | 1.74 | yes |
| 30-19 | 3.2 | 10 | 1.86 | yes |
| 30-19 | 3.2 | 10 | 2.18 | yes |
| 30-19 | 3.2 | 24 | 0.46 | no |
| 30-19 | 3.2 | 24 | 0.51 | no |
| 30-19 | 3.2 | 24 | 0.74 | no |
| 30-19 | 3.2 | 24 | 0.43 | yes |
| 30-19 | 3.2 | 24 | 1.22 | yes |
| 30-19 | 3.2 | 24 | 3.10 | yes |
| 30-19 | 3.2 | 24 | 3.86 | yes |
| 30-19 | 20.9 | 4 | 0.39 | no |
| 30-19 | 20.9 | 4 | 0.40 | no |
| 30-19 | 20.9 | 4 | 0.54 | yes |
| 30-19 | 20.9 | 4 | 0.58 | yes |
| 30-19 | 20.9 | 4 | 0.78 | yes |
| 30-19 | 20.9 | 4 | 0.80 | yes |
| 30-19 | 20.9 | 4 | 0.86 | yes |
| 30-19 | 20.9 | 4 | 0.89 | yes |
| 30-19 | 20.9 | 4 | 0.98 | yes |
| 30-19 | 20.9 | 10 | 0.49 | yes |
| 30-19 | 20.9 | 10 | 1.33 | yes |
| 30-19 | 20.9 | 24 | 0.59 | no |
| 30-19 | 20.9 | 24 | 0.63 | no |
| 30-19 | 20.9 | 24 | 1.28 | yes |
| 30-19 | 20.9 | 24 | 2.87 | yes |
| 30-19 | 20.9 | 24 | 3.86 | yes |
| 30-19 | 20.9 | 24 | 3.88 | yes |
| 30-19 | 20.9 | 24 | 3.90 | yes |
| 30-19 | 20.9 | 24 | 4.79 | yes |
| 30-19 | 20.9 | 24 | 5.93 | yes |
| 31-05 | 2.8 | 4 | 0.40 | no |
| 31-05 | 2.8 | 4 | 0.53 | no |
| 31-05 | 2.8 | 4 | 0.62 | no |
| 31-05 | 2.8 | 4 | 0.66 | no |
| 31-05 | 2.8 | 4 | 0.69 | no |
| 31-05 | 2.8 | 4 | 0.70 | no |
| 31-05 | 2.8 | 4 | 0.73 | no |
| 31-05 | 2.8 | 4 | 0.40 | yes |
| 31-05 | 2.8 | 4 | 0.43 | yes |
| 31-05 | 2.8 | 4 | 0.52 | yes |
| 31-05 | 2.8 | 4 | 0.57 | yes |
| 31-05 | 2.8 | 4 | 0.61 | yes |
| 31-05 | 2.8 | 4 | 0.71 | yes |
| 31-05 | 2.8 | 4 | 0.83 | yes |
| 31-05 | 2.8 | 4 | 0.95 | yes |
| 31-05 | 2.8 | 15 | 1.74 | no |
| 31-05 | 2.8 | 15 | 0.52 | yes |
| 31-05 | 2.8 | 15 | 0.54 | yes |
| 31-05 | 2.8 | 15 | 1.77 | yes |
| 31-05 | 2.8 | 15 | 2.66 | yes |
| 31-05 | 2.8 | 25 | 0.41 | no |
| 31-05 | 2.8 | 25 | 5.39 | no |
| 31-05 | 2.8 | 25 | 5.69 | yes |
| 31-05 | 2.8 | 25 | 5.80 | yes |
| 31-05 | 2.8 | 25 | 6.27 | yes |
| 31-05 | 15.8 | 4 | 0.49 | no |
| 31-05 | 15.8 | 4 | 0.53 | no |
| 31-05 | 15.8 | 4 | 0.54 | no |
| 31-05 | 15.8 | 4 | 0.54 | no |
| 31-05 | 15.8 | 4 | 0.64 | no |
| 31-05 | 15.8 | 4 | 0.58 | yes |
| 31-05 | 15.8 | 15 | 0.42 | no |
| 31-05 | 15.8 | 15 | 0.77 | no |
| 31-05 | 15.8 | 15 | 0.92 | no |
| 31-05 | 15.8 | 15 | 1.12 | no |
| 31-05 | 15.8 | 15 | 1.72 | no |
| 31-05 | 15.8 | 15 | 1.98 | no |
| 31-05 | 15.8 | 25 | 4.30 | no |
| 31-05 | 15.8 | 25 | 5.04 | no |
| 31-05 | 15.8 | 25 | 5.48 | no |
| 32-02 | 3.5 | 0 | 0.36 | yes |
| 32-02 | 3.5 | 4 | 0.45 | no |
| 32-02 | 3.5 | 4 | 0.49 | no |
| 32-02 | 3.5 | 4 | 0.61 | no |
| 32-02 | 3.5 | 4 | 0.79 | no |
| 32-02 | 3.5 | 4 | 0.90 | no |
| 32-02 | 3.5 | 12 | 0.65 | no |
| 32-02 | 3.5 | 12 | 1.81 | no |
| 32-02 | 3.5 | 12 | 1.00 | yes |
| 32-02 | 3.5 | 12 | 1.12 | yes |
| 32-02 | 3.5 | 12 | 1.34 | yes |
| 32-02 | 3.5 | 12 | 1.52 | yes |
| 32-02 | 3.5 | 24 | 4.98 | no |
| 32-02 | 3.5 | 24 | 5.96 | no |
| 32-02 | 3.5 | 24 | 7.24 | no |
| 32-02 | 3.5 | 24 | 5.84 | yes |

Table S5: Table with single specimen enrichments derived from NanoSIMS analysis for Trichodesmium.

| Station | Depth | Sampling time | ^15^N [atom %] |
| --- | --- | --- | --- |
| 30-19 | 20.9 | 4 | 0.43 |
| 30-19 | 20.9 | 4 | 0.46 |
| 30-19 | 20.9 | 4 | 0.49 |
| 30-19 | 20.9 | 4 | 0.50 |
| 30-19 | 20.9 | 4 | 0.52 |
| 30-19 | 20.9 | 4 | 0.52 |
| 30-19 | 20.9 | 4 | 0.53 |
| 30-19 | 20.9 | 4 | 0.54 |
| 30-19 | 20.9 | 4 | 0.54 |
| 30-19 | 20.9 | 4 | 0.61 |
| 30-19 | 20.9 | 4 | 0.80 |
| 30-19 | 20.9 | 10 | 0.51 |
| 30-19 | 20.9 | 10 | 0.51 |
| 30-19 | 20.9 | 10 | 0.52 |
| 30-19 | 20.9 | 10 | 0.55 |
| 30-19 | 20.9 | 10 | 0.58 |
| 30-19 | 20.9 | 10 | 0.62 |
| 30-19 | 20.9 | 10 | 0.63 |
| 30-19 | 20.9 | 10 | 0.68 |
| 30-19 | 20.9 | 10 | 0.72 |
| 30-19 | 20.9 | 10 | 0.97 |
| 30-19 | 20.9 | 10 | 1.40 |
| 30-19 | 20.9 | 24 | 0.59 |
| 30-19 | 20.9 | 24 | 0.70 |
| 30-19 | 20.9 | 24 | 0.88 |
| 30-19 | 20.9 | 24 | 1.27 |
| 30-19 | 20.9 | 24 | 1.36 |
| 30-19 | 20.9 | 24 | 3.10 |
| 30-19 | 3.2 | 0 | 0.36 |
| 30-19 | 3.2 | 0 | 0.37 |
| 30-19 | 3.2 | 0 | 0.38 |
| 30-19 | 3.2 | 0 | 0.38 |
| 30-19 | 3.2 | 0 | 0.37 |
| 30-19 | 3.2 | 0 | 0.38 |
| 30-19 | 3.2 | 0 | 0.37 |
| 30-19 | 3.2 | 0 | 0.38 |
| 30-19 | 3.2 | 4 | 0.40 |
| 30-19 | 3.2 | 4 | 0.48 |
| 30-19 | 3.2 | 4 | 0.48 |
| 30-19 | 3.2 | 4 | 0.52 |
| 30-19 | 3.2 | 4 | 0.55 |
| 30-19 | 3.2 | 4 | 0.55 |
| 30-19 | 3.2 | 4 | 0.57 |
| 30-19 | 3.2 | 4 | 0.57 |
| 30-19 | 3.2 | 4 | 0.59 |
| 30-19 | 3.2 | 4 | 0.60 |
| 30-19 | 3.2 | 4 | 0.68 |
| 30-19 | 3.2 | 4 | 0.77 |
| 30-19 | 3.2 | 10 | 0.43 |
| 30-19 | 3.2 | 10 | 0.47 |
| 30-19 | 3.2 | 10 | 0.53 |
| 30-19 | 3.2 | 10 | 0.57 |
| 30-19 | 3.2 | 10 | 0.58 |
| 30-19 | 3.2 | 10 | 0.58 |
| 30-19 | 3.2 | 10 | 0.61 |
| 30-19 | 3.2 | 10 | 0.61 |
| 30-19 | 3.2 | 10 | 0.65 |
| 30-19 | 3.2 | 10 | 0.77 |
| 30-19 | 3.2 | 10 | 0.78 |
| 30-19 | 3.2 | 10 | 0.83 |
| 30-19 | 3.2 | 10 | 0.83 |
| 30-19 | 3.2 | 10 | 0.84 |
| 30-19 | 3.2 | 10 | 0.87 |
| 30-19 | 3.2 | 10 | 0.88 |
| 30-19 | 3.2 | 10 | 0.95 |
| 30-19 | 3.2 | 10 | 0.99 |
| 30-19 | 3.2 | 10 | 1.58 |
| 30-19 | 3.2 | 24 | 0.59 |
| 30-19 | 3.2 | 24 | 0.69 |
| 30-19 | 3.2 | 24 | 0.78 |
| 30-19 | 3.2 | 24 | 0.85 |
| 30-19 | 3.2 | 24 | 0.86 |
| 30-19 | 3.2 | 24 | 0.90 |
| 30-19 | 3.2 | 24 | 0.92 |
| 30-19 | 3.2 | 24 | 0.93 |
| 30-19 | 3.2 | 24 | 0.94 |
| 30-19 | 3.2 | 24 | 0.96 |
| 30-19 | 3.2 | 24 | 1.03 |
| 30-19 | 3.2 | 24 | 1.07 |
| 30-19 | 3.2 | 24 | 1.13 |
| 30-19 | 3.2 | 24 | 1.17 |
| 30-19 | 3.2 | 24 | 1.18 |
| 30-19 | 3.2 | 24 | 1.19 |
| 30-19 | 3.2 | 24 | 3.77 |
| 31-05 | 15.8 | 0 | 0.36 |
| 31-05 | 15.8 | 0 | 0.36 |
| 31-05 | 15.8 | 0 | 0.36 |
| 31-05 | 15.8 | 4 | 0.43 |
| 31-05 | 15.8 | 4 | 0.40 |
| 31-05 | 15.8 | 4 | 0.51 |
| 31-05 | 15.8 | 4 | 0.46 |
| 31-05 | 15.8 | 15 | 0.48 |
| 31-05 | 15.8 | 15 | 0.52 |
| 31-05 | 15.8 | 25 | 0.42 |
| 31-05 | 15.8 | 25 | 0.45 |
| 31-05 | 15.8 | 25 | 0.49 |
| 31-05 | 15.8 | 25 | 1.77 |
| 31-05 | 15.8 | 25 | 2.00 |
| 31-05 | 2.8 | 0 | 0.36 |
| 31-05 | 2.8 | 0 | 0.36 |
| 31-05 | 2.8 | 4 | 0.43 |
| 31-05 | 2.8 | 15 | 0.40 |
| 31-05 | 2.8 | 15 | 0.40 |
| 31-05 | 2.8 | 15 | 0.40 |
| 31-05 | 2.8 | 15 | 0.41 |
| 31-05 | 2.8 | 15 | 0.41 |
| 31-05 | 2.8 | 15 | 0.43 |
| 31-05 | 2.8 | 15 | 0.44 |
| 31-05 | 2.8 | 15 | 0.47 |
| 31-05 | 2.8 | 15 | 0.62 |
| 31-05 | 2.8 | 15 | 0.77 |
| 31-05 | 2.8 | 15 | 0.79 |
| 31-05 | 2.8 | 15 | 0.86 |
| 31-05 | 2.8 | 15 | 0.90 |
| 31-05 | 2.8 | 25 | 0.41 |
| 31-05 | 2.8 | 25 | 0.48 |
| 31-05 | 2.8 | 25 | 0.65 |
| 31-05 | 2.8 | 25 | 0.76 |
| 31-05 | 2.8 | 25 | 0.80 |
| 31-05 | 2.8 | 25 | 0.81 |
| 32-02 | 3.5 | 0 | 0.36 |
| 32-02 | 3.5 | 0 | 0.36 |
| 32-02 | 3.5 | 0 | 0.36 |
| 32-02 | 3.5 | 0 | 0.35 |
| 32-02 | 3.5 | 0 | 0.37 |
| 32-02 | 3.5 | 0 | 0.37 |
| 32-02 | 3.5 | 0 | 0.37 |
| 32-02 | 3.5 | 0 | 0.36 |
| 32-02 | 3.5 | 0 | 0.36 |
| 32-02 | 3.5 | 0 | 0.36 |
| 32-02 | 3.5 | 0 | 0.37 |
| 32-02 | 3.5 | 0 | 0.36 |
| 32-02 | 3.5 | 0 | 0.36 |
| 32-02 | 3.5 | 0 | 0.36 |
| 32-02 | 3.5 | 24 | 1.36 |
| 32-02 | 3.5 | 24 | 0.59 |
| 32-02 | 3.5 | 24 | 1.84 |
| 32-02 | 3.5 | 24 | 4.90 |
| 32-02 | 3.5 | 24 | 1.68 |
| 32-02 | 3.5 | 24 | 0.40 |

Table S6: Table with single specimen enrichments derived from NanoSIMS analysis for Chaetoceros and Rhizosolenia-Richelia.

| Station | Depth | Sampling time | 15N [atom %] | Species |
| --- | --- | --- | --- | --- |
| 30 | 4.9 | 0 | 0.38 | *Chaetoceros spp.* |
| 30 | 4.9 | 0 | 0.39 | *Chaetoceros spp.* |
| 30 | 4.9 | 0 | 0.38 | *Chaetoceros spp.* |
| 30 | 4.9 | 24 | 2.46 | *Chaetoceros spp.* |
| 30 | 4.9 | 24 | 2.95 | *Chaetoceros spp.* |
| 31 | 4.08 | 4 | 0.58 | *Chaetoceros spp.* |
| 31 | 4.08 | 15 | 0.41 | *Chaetoceros spp.* |
| 31 | 4.08 | 15 | 0.41 | *Chaetoceros spp.* |
| 31 | 4.08 | 15 | 0.43 | *Chaetoceros spp.* |
| 31 | 4.08 | 25 | 0.47 | *Chaetoceros spp.* |
| 31 | 4.08 | 25 | 2.73 | *Chaetoceros spp.* |
| 30 | 4.9 | 10 | 1.29 | *Chaetoceros spp.* |
| 30 | 4.9 | 10 | 1.15 | *Chaetoceros spp.* |
| 30 | 4.9 | 10 | 1.34 | *Chaetoceros spp.* |
| 30 | 20.9 | 24 | 3.58 | *Rhizosolenia-Richelia* |
| 30 | 20.9 | 24 | 3.74 | *Rhizosolenia-Richelia* |
| 30 | 4.9 | 4 | 0.83 | *Rhizosolenia-Richelia* |
| 31 | 15.8 | 25 | 1.33 | *Rhizosolenia-Richelia* |
| 31 | 15.8 | 25 | 1.33 | *Rhizosolenia-Richelia* |
| 31 | 4.08 | 15 | 0.61 | *Rhizosolenia-Richelia* |
| 32 | 4.22 | 0 | 0.35 | *Rhizosolenia-Richelia* |
| 32 | 4.22 | 0 | 0.37 | *Rhizosolenia-Richelia* |

**Supplementary References**

1. Grasshoff K, Kremling K, Erhardt M. *Methods of seawater analysis*, Weinheim: Wiley-VCH, 1999.

2. Dabundo R, Lehmann MF, Treibergs L *et al.* The contamination of commercial ^15^n_2_ gas stocks with ^15^n–labeled nitrate and ammonium and consequences for nitrogen fixation measurements. *PLoS ONE*. 2014;**9**:e110335 <https://doi.org/doi:10.1371/journal.pone.0110335>

3. Zhang S, Fang Y, Xi D. Adaptation of micro-diffusion method for the analysis of 15n natural abundance of ammonium in samples with small volume. *Rapid Communication in Mass Spectrometry*. 2015;**29**:1297-306 <https://doi.org/10.1002/rcm.7224>

4. Holmes RM, McClelland JW, Sigman DM *et al.* Measuring ^15^n-nh_4_^+^ in marine, estuarine and fresh waters: An adaptation of the ammonia diffusion method for samples with low ammonium concentrations. *Marine Chemistry*. 1998;**60**:235-43

5. White AE, Granger J, Selden C *et al.* A critical review of the 15n2 tracer method to measure diazotrophic production in pelagic ecosystems. *Limnology and Oceanography: Methods*. 2020;**18**:129-47 <https://doi.org/10.1002/lom3.10353>

6. Wannicke N, Benavides M, Dalsgaard T *et al.* New perspectives on nitrogen fixation measurements using 15n2 gas. *Frontiers in Marine Science*. 2018;**5** <https://doi.org/10.3389/fmars.2018.00120>

7. Schvarcz CR, Wilson ST, Caffin M *et al.* Overlooked and widespread pennate diatom-diazotroph symbioses in the sea. *Nature Communications*. 2022;**13**:799 <https://doi.org/10.1038/s41467-022-28065-6>

8. Tschitschko B, Esti M, Philippi M *et al.* Rhizobia–diatom symbiosis fixes missing nitrogen in the ocean. *Nature*. 2024;**630**:899-904 <https://doi.org/10.1038/s41586-024-07495-w>

9. Nakayama T, Inagaki Y. Genomic divergence within non-photosynthetic cyanobacterial endosymbionts in rhopalodiacean diatoms. *Scientific Reports*. 2017;**7**:13075 <https://doi.org/10.1038/s41598-017-13578-8>

10. Gibson RA, Stephen FC. Valve structure in mastogloia rostrata with a comparison of intercalary band internal construction in two dissimilar diatom species. *Cryptogamie, Algologie*. 1985;**6**:13-24 <https://doi.org/10.5962/p.309117>

11. Simonsen R. The diatom plankton of the indian ocean expedition of r/v" meteor" 1964-1965. *Meteor Forschungsergebnisse: Reihe D, Biologie*. 1974;**19**:1-107

12. Sterrenburg FA, Tiffany MA, Hinz F *et al.* Seven new species expand the morphological spectrum of haslea. A comparison with gyrosigma and pleurosigma (bacillariophyta). *Phytotaxa*. 2015;**207**:143-62 <https://doi.org/10.11646/phytotaxa.207.2.1>

13. Kryk A, Ba˛k M, Gorecka E *et al.* Marine diatom assemblages of the nosy be island coasts, nw madagascar: Species composition and biodiversity using molecular and morphological taxonomy. *Systematics and Biodiversity*. 2020;**18**:161-80 <https://doi.org/10.1080/14772000.2019.1696420>

14. Pennesi C, Poulin M, De Stefano M *et al.* New insights to the ultrastructure of some marine mastogloia species section sulcatae (bacillariophyceae), including m. Neoborneensis sp. Nov. *Phycologia*. 2011;**50**:548-62 <https://doi.org/10.2216/10-39.1>

15. Scharek R, Latasa M, Karl DM *et al.* Temporal variations in diatom abundance and downward vertical flux in the oligotrophic north pacific gyre. *Deep-Sea Research I*. 1999;**46**:1051-75

16. Polerecky L, Adam B, Milucka J *et al.* Look@ nanosims–a tool for the analysis of nanosims data in environmental microbiology. *Environmental microbiology*. 2012;**14**:1009-23

17. Montoya JP, Carpenter EJ, Capone DG. Nitrogen fixation and nitrogen isotope abundance in zooplankton of the oligotrophic north atlantic. *Limnol Oceanogr*. 2002;**47**:1617-28

18. Benavides M, Bronk DA, Agawin NSR *et al.* Longitudinal variability of size-fractionated n2 fixation and don release rates along 24.5°n in the subtropical north atlantic. *Journal of Geophysical Research: Oceans*. 2013;**118**:3406-15 <https://doi.org/10.1002/jgrc.20253>

19. Kopylova E, Noé L, Touzet H. Sortmerna: Fast and accurate filtering of ribosomal rnas in metatranscriptomic data. *Bioinformatics*. 2012;**28**:3211-17 <https://doi.org/10.1093/bioinformatics/bts611>

20. Bushmanova E, Antipov D, Lapidus A *et al.* Rnaspades: A de novo transcriptome assembler and its application to rna-seq data. *GigaScience*. 2019;**8** <https://doi.org/10.1093/gigascience/giz100>

21. Li B, Dewey CN. Rsem: Accurate transcript quantification from rna-seq data with or without a reference genome. *BMC Bioinformatics*. 2011;**12**:323 <https://doi.org/10.1186/1471-2105-12-323>

22. Seemann T. Prokka: Rapid prokaryotic genome annotation. *Bioinformatics*. 2014;**30**:2068-69 <https://doi.org/10.1093/bioinformatics/btu153>

23. Menzel P, Ng KL, Krogh A. Fast and sensitive taxonomic classification for metagenomics with kaiju. *Nature Communications*. 2016;**7**:11257 <https://doi.org/10.1038/ncomms11257>

24. Moynihan MA, Furbo Reeder C. Nifhdada2 github repository. Report. 2023
